# Impact of delayed processing of positive blood cultures on organism detection: a prospective multi-centre study

**DOI:** 10.1186/s12879-022-07504-1

**Published:** 2022-06-04

**Authors:** Tamalee Roberts, Arjun Chandna, Wanitda Watthanaworawit, Areerat Thaiprakong, Sona Soeng, Manivone Simmalavong, Phonelavanh Phoumin, Weerawut Saengchun, Nongyao Khatta, Pattaraporn Hinfonthong, Napaporn Kaewpundoem, Sue J. Lee, Carlo Perrone, Ben Amos, Paul Turner, Elizabeth A. Ashley, Clare L. Ling

**Affiliations:** 1 Lao-Oxford-Mahosot Hospital-Wellcome Trust Research Unit, Microbiology Laboratory, Mahosot Hospital, Lao People’s Democratic Republic, Vientiane, Laos; 2 Centre for Tropical Medicine and Global Health, Nuffield Department of Medicine, University of Oxford, Oxford, UK; 3 Cambodia Oxford Medical Research Unit, Angkor Hospital for Children, Siem Reap, Cambodia; 4 Shoklo Malaria Research Unit, Mahidol-Oxford Tropical Medicine Research Unit, Faculty of Tropical Medicine, Mahidol University, Mae Sot, Thailand; 5 Mahidol-Oxford Tropical Medicine Research Unit, Faculty of Tropical Medicine, Mahidol University, Phaya Thai, Bangkok, Thailand; 6 Department of Microbiology, Chiangrai Prachanukroh Hospital, Chiangrai, Thailand; 7 Independent consultant, Falmouth, Cornwall UK

**Keywords:** Blood culture, Bloodstream infection, Low- and middle-income country, Southeast Asia, Diagnostics

## Abstract

**Background:**

Blood cultures remain the gold standard investigation for the diagnosis of bloodstream infections. In many locations, quality-assured processing of positive blood cultures is not possible. One solution is to incubate blood cultures locally, and then transport bottles that flag positive to a central reference laboratory for organism identification and antimicrobial susceptibility testing. However, the impact of delay between the bottle flagging positive and subsequent sub-culture on the viability of the isolate has received little attention.

**Methods:**

This study evaluated the impact of delays to sub-culture (22 h to seven days) in three different temperature conditions (2–8 °C, 22–27 °C and 35 ± 2 °C) for bottles that had flagged positive in automated detection systems using a mixture of spiked and routine clinical specimens. Ninety spiked samples for five common bacterial causes of sepsis (*Escherichia coli*, *Haemophilus influenzae, Staphylococcus aureus, Streptococcus agalactiae* and *Streptococcus pneumoniae*) and 125 consecutive positive clinical blood cultures were evaluated at four laboratories located in Cambodia, Lao PDR and Thailand. In addition, the utility of transport swabs for preserving organism viability was investigated.

**Results:**

All organisms were recoverable from all sub-cultures in all temperature conditions with the exception of *S. pneumoniae*, which was less likely to be recoverable after longer delays (> 46–50 h), when stored in hotter temperatures (35 °C), and from BacT/ALERT when compared with BACTEC blood culture bottles. Storage of positive blood culture bottles in cooler temperatures (22–27 °C or below) and the use of Amies bacterial transport swabs helped preserve viability of *S. pneumoniae*.

**Conclusions:**

These results have practical implications for the optimal workflow for blood culture bottles that have flagged positive in automated detection systems located remotely from a central processing laboratory, particularly in tropical resource-constrained contexts.

**Supplementary Information:**

The online version contains supplementary material available at 10.1186/s12879-022-07504-1.

## Background

There is an increasing trend towards centralisation of hospital laboratory services. In high-income settings this is often driven by a desire to rationalise provision of specialist tests and perceived benefits of ‘economies of scale’ [1]. In rural and remote regions of low- and middle-income countries (LMICs) absence of the necessary infrastructure and resources may mean that centralisation may be the only viable option to provide certain quality-assured laboratory services.

Blood cultures remain the investigation of choice for the diagnosis of bloodstream infections (BSIs). Commercially produced bottles and automated detection systems have been well validated and are used by many laboratories. Two possible strategies for centralisation can be considered: transfer of inoculated bottles prior to incubation or local incubation of the blood culture (BC) bottles followed by transfer only of those bottles that flag positive. In comparison, the latter reduces the time between collection and primary incubation, time to detection of positivity, and allows a Gram stain to be performed locally to inform patient care. Further it reduces the number of bottles that have to be transferred simplifying logistics.

Several studies have demonstrated the negative impact of delay between sample collection and primary incubation on the rates of pathogen recovery [2–4]. However, little attention has been paid to the effect of delay to sub-culture once a bottle has already flagged positive. Positive BC bottles can contain between 2 × 10^7^ and 7 × 10^9^ CFU/ml of bacteria [5, 6], approximately a 7 to 9-fold increase compared with the concentration present at the time of inoculation [7, 8]. Hence, delays to sub-culture may impact organism viability due to exhaustion of the nutrients available in the culture media required to sustain bacterial growth. Delay to sub-culture can result in autolysis of *Streptococcus pneumoniae* in the culture media [9]. One study showed that a delay of 24 h at room temperature after primary incubation had an effect on rapid identification methods with a difference demonstrated between Gram-positive and Gram-negative organisms [10]. These studies did not look at the impact of different delay times or environmental temperatures. In the tropical resource-limited environments common in many LMICs, delays to sub-culture after primary incubation may often exceed 24 h at high ambient temperatures, with poor road infrastructure and weather conditions increasing transit times.

This study investigated the impact of different delays to sub-culture (22–26 h, 30–34 h, 46–50 h and seven days) in three different temperature conditions (2–8 °C, 22–27 °C and 35 ± 2 °C), for spiked BC bottles that had flagged positive in an automated detection system (BacT/ALERT or BACTEC). Secondly, the impact of delay to sub-culture at different time points on clinical BC bottles was evaluated. In addition, the utility of using Amies transport swabs for the safe transport of positive BC broth was investigated.

## Materials and methods

### Study sites

The study took place at four sites in Southeast Asia: Mahosot Hospital Microbiology Laboratory affiliated with the Lao-Oxford-Mahosot Hospital-Wellcome Trust Research Unit (LOMWRU) in Vientiane, Lao PDR; the Cambodia Oxford Medical Research Unit (COMRU) in Siem Reap, Cambodia; the Shoklo Malaria Research Unit (SMRU) in Mae Sot, Thailand; and the Department of Microbiology, Chiangrai Prachanukroh Hospital (CPH), Chiangrai, Thailand. Mahosot Hospital is an ~ 400-bed government hospital providing primary to tertiary care and admitting ~ 2000 patients/month [11]. COMRU supports the diagnostic microbiology service at Angkor Hospital for Children, an ~ 100-bed non-governmental paediatric hospital [12]. SMRU supports several clinics located along the Thailand-Myanmar border that provide healthcare for marginalised populations [13]. Chiangrai Prachanukroh Hospital is an ~ 800-bed government hospital providing primary to tertiary care and admitting ~ 4,000 patients/month [14].

### Phase one

Human packed red blood cells (PRBC) approaching expiry were acquired from local blood banks and spiked with a target organism (one bag per organism per site) – *Escherichia coli*, *Haemophilus influenzae, Staphylococcus aureus, Streptococcus agalactiae* and *Streptococcus pneumoniae*. These organisms were selected to reflect common causes of adult and paediatric BSIs. Stored clinical isolates from each site, previously identified using standard identification methods (biochemical tests [including Triple Sugar Iron, urease, oxidase, catalase, slide coagulase and Sulphide Indole Motility] and/or API and/or MALDI-TOF MS), were used to imitate real-world BSIs in preference to ATCC reference strains subjected to multiple passages that may have resulted in the selection of fitter organisms. *S. pneumoniae* and *S. agalactiae* were processed at LOMWRU, *H. influenzae, S. pneumoniae* and *E. coli* at COMRU, and *S. aureus* at SMRU. Due to the potential impact of the different bottles and an *a priori* assumption that *S. pneumoniae* would be the organism most susceptible to delayed sub-culture, both COMRU and LOMWRU processed this organism.

Bacterial colonies less than 24 h old from sheep / goat blood agar (chocolate agar for bottles spiked with *H. influenzae*) were suspended in normal saline to create a solution equivalent to a 1 McFarland standard (approximately 3 × 10^8^ CFU/ml). This was diluted using 10-fold dilutions to approximately 300 CFU/ml. A 5-fold dilution was then made to give a concentration of ~ 60 CFU/ml. A final 1 in 10 dilution of the suspension with the PRBCs was made to obtain a final concentration of ~ 6 CFU/ml, 1 ml of which was inoculated into each of 15 BC bottles (five replicates for each of the three temperature conditions). Six CFU/ml was chosen to approximate the concentration of bacteria present in human BSIs [7, 8]. The Miles and Misra method was used to confirm the actual concentration inoculated [15].

**Fig. 1:**
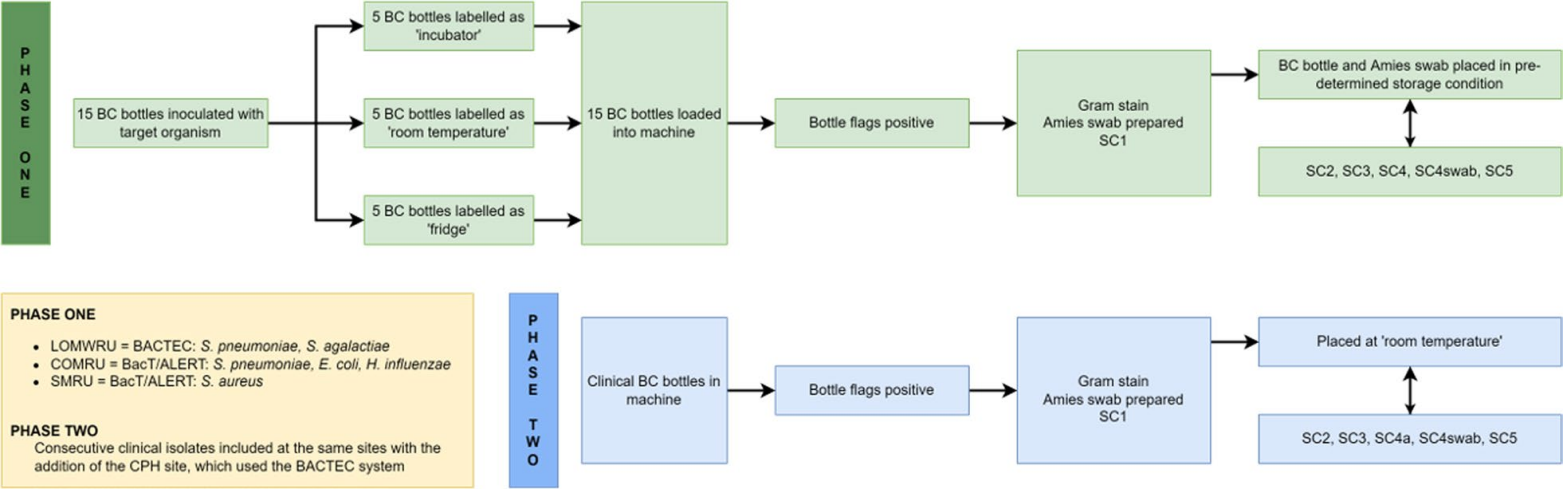
Study flowchart for Phase One and Phase Two. Phase One represented in green and Phase Two represented in blue. BC = blood culture, COMRU = Cambodia Oxford Medical Research Unit; CPH = Chiangrai Provincial Hospital; LOMWRU = Lao-Oxford-Mahosot Hospital-Wellcome Trust Research Unit; SMRU = Shoklo Malaria Research Unit; SC = sub-culture; SC1 = 0–4 h; SC2 = 22–26 h; SC3 = 30–34 h; SC4 = 46–50 h; SC4 swab = Amies transport swab stored for 46–50 h; SC5 = 7 days

Inoculated bottles were incubated in the automated detection system until they flagged positive or for at least five days, whichever occurred sooner. PEDS Plus/F bottles (Becton Dickinson, NJ, USA) were used with the automated BD BACTEC FX system at LOMWRU, and PF Plus bottles (bioMérieux, Marcy-l’Étoile, France) were used with the automated BacT/ALERT 3D system at COMRU and SMRU (Fig. 1).

When a bottle flagged positive, 2–4 drops of BC broth were sub-cultured (SC1) onto sheep / goat blood agar (chocolate agar for *H. influenzae*) using the streak plate technique, and 5–7 drops of broth dropped into a test tube, which was used to saturate an Amies bacterial transport swab (Copan eSwab, Brescia, Italy for LOMWRU and SMRU [and CPH for Phase Two], and ThermoFisher BactiSwab, MA, USA for COMRU). Agar plates were incubated in aerobic conditions (and with 5% CO_2_ for *H. influenzae* and *S. pneumoniae*) at 35 ± 2 °C for 24 h, extended to 48 h if there was no growth after 24 h. The bottles and swabs were stored at the pre-specified temperatures (2–8 °C [fridge], 22–27 °C [incubator or monitored air-conditioned room temperature, RT] and 35 ± 2 °C [incubator]) to simulate a range of feasible real-world transit temperatures. Bottles were then sub-cultured at approximately 22–26 h (SC2), 30–34 h (SC3), 46–50 h (SC4) and seven days (SC5) after removal from the machine. The Amies transport swab was sub-cultured once at 46–50 h (SC4 swab). Growth of the target organism on sub-culture was confirmed by Gram stain and colony morphology (conventional biochemicals and/or MALDI-TOF MS [VITEK MS, bioMérieux] were used in the case of uncertainty), and semi-quantified using standard techniques (No growth; Light growth = growth in quadrant 1 and < 10 colonies in quadrant 2 [± or +]; Moderate growth = growth in quadrant 2 and < 10 colonies in quadrant 3 [2+]; Heavy growth = growth in quadrant 3 ± quadrant 4 [3 + or 4+]).

### Phase two

Consecutive clinical BC bottles from children and adults that flagged positive on the respective automated detection systems at LOMWRU, COMRU and SMRU, and the BACTEC at CPH were included. In addition to the bottles used for Phase One, BD BACTEC Plus aerobic bottles were used with the BACTEC system at CPH and LOMWRU and BACT/ALERT FA PLUS were used with the BacT/ALERT system at SMRU. Duplicate patient samples were not excluded. Positive bottles were sub-cultured onto the appropriate agar according to local standard operating procedures (SOP) and incubated overnight as in Phase One. Approximately 5–7 drops were used to saturate an Amies bacterial transport swab as above. The bottles and swabs were stored on the bench (monitored air-conditioned room; Additional file 1: Fig. S1) and sub-cultured as in Phase One (Fig. 1). This temperature was selected balancing the results from Phase One with what might be practical in resource-constrained tropical environments. Growth on the first sub-culture was identified phenotypically using conventional biochemicals and/or MALDI-TOF MS, according to local SOPs. Sequential sub-culture growth was confirmed as the original isolate based on colonial morphology (or further testing if unclear), and growth semi-quantified as described above. Agar plates were incubated for 24 h, extended to 48 h if there was no growth after 24 h.

Ambient temperature was monitored continuously at all four sites during Phase Two using the TinyTag Transit 2 (Gemini Data Loggers, UK) at COMRU and LOMWRU, MicroLite (Fourtec, USA) at SMRU and Escort iMiniPlus (Escort Data Logging Systems, Germany) at CPH.

### Quality control

All sites complete media quality control following their internal quality control system. All sites participate in quality assurance programs: LOMWRU participates in the UK National External Quality Assurance Scheme; COMRU participates in the Pacific Pathology Training Centre Microbiology Quality Assurance Programme; SMRU participates in the Thailand Clinical Microbiology Proficiency Testing Scheme, Division of Proficiency Testing, Department of Medical Science, Ministry of Public Health; and CPH participates in the Thailand Medical Technology Council accreditation program. Results are reported following the MICRO Checklist [16].

### Statistical analysis

All data were entered into Microsoft Excel (Richmond, WA). For Phase One, growth of *S. pneumoniae* between different sub-cultures and temperature conditions was compared using the Wilcoxon matched-pairs signed-rank test. For Phase Two, semi-quantitative growth between *Streptococcus* spp. and all other organisms isolated was compared using the Fisher’s exact test. P-values less than 0.05 were considered statistically significant. Analysis was performed using Stata version 16.1 (StataCorp, College Station, TX).

## Results

### Phase one

#### Inoculum

Bottles were confirmed to have been inoculated with PRBCs containing 1.2–10.6 CFU/ml (median 5.8 CFU/ml) of bacteria. All incubated BC bottles flagged positive in the automated detection systems except one bottle in the BacT/ALERT system that had been inoculated with *S. pneumoniae.* The median time to sub-culture after a bottle flagged positive was 5 h 6 min (interquartile range [IQR] = 29 min to 5 h 57 min). The median CFU/ml, time to positivity, time to removing the bottle from the automated system and time to sub-culture for each organism is presented in Additional file 1: Table S1.

#### Sub-culture results: blood culture bottles (spiked samples)

The expected organism was isolated from SC1 for all positive bottles. *S. agalactiae*, *S. aureus, H. influenzae* and *E. coli* grew at every time point for all temperature conditions. All *S. pneumoniae* sub-cultures grew for every time point and temperature condition using the bottles loaded in the BACTEC system. However, there were varying results using the BacT/ALERT system; for bottles stored at 35 ± 2 °C *S. pneumoniae* grew from 4/5 bottles at SC2, 1/5 bottles at SC3 and no bottles at SC4 and SC5; for bottles stored at RT there was growth from 2/5 bottles at SC4 and no bottles at SC5; and for bottles stored at 2–8 °C there was growth from all bottles at all sub-cultures (Table 1). Fewer bottles resulted in growth for *S. pneumoniae* for all SCs compared to SC1 for the bottles stored at 35 ± 2 °C, and also for SC4 and SC5 compared to SC1 at RT. (Additional file 1: Table S2).

**Table 1:** Number of sub-cultures with growth from blood culture bottles spiked with different organisms and stored for different times at different temperatures after flagging positive

Organism (blood culture system, laboratory site)	SC1 (0–6 h)	Storage condition	SC2 (22–26 h)	SC3 (30–34 h)	SC4 (46–50 h)	SC4 swab* (46–50 h)	SC5 (7 days)
*S. agalactiae* (BACTEC, LOMWRU)	15/15	Fridge	5/5	5/5	5/5	5/5	5/5
		RT	5/5	5/5	5/5	5/5	5/5
		Incubator	5/5	5/5	5/5	5/5	5/5
*S. pneumoniae* (BACTEC, LOMWRU)	15/15	Fridge	5/5	5/5	5/5	5/5	5/5
		RT	5/5	5/5	5/5	5/5	5/5
		Incubator	5/5	5/5	5/5	5/5	5/5
*S. pneumoniae* (BacT/ALERT, COMRU)	14/14	Fridge	4/4	4/4	4/4	4/4	4/4
		RT	5/5	5/5	2/5	5/5	0/5
		Incubator	4/5	1/5	0/5	5/5	0/5
*S. aureus* (BacT/ALERT, SMRU)	15/15	Fridge	5/5	5/5	5/5	5/5	5/5
		RT	5/5	5/5	5/5	5/5	5/5
		Incubator	5/5	5/5	5/5	5/5	5/5
*H. influenzae* (BacT/ALERT, COMRU)	15/15	Fridge	5/5	5/5	5/5	5/5	5/5
		RT	5/5	5/5	5/5	5/5	5/5
		Incubator	5/5	5/5	5/5	2/5	5/5
*E. coli* (BacT/ALERT, COMRU)	15/15	Fridge	5/5	5/5	5/5	5/5	5/5
		RT	5/5	5/5	5/5	5/5	5/5
		Incubator	5/5	5/5	5/5	5/5	5/5

SC = sub-culture (duration of time stored after flagging positive), Fridge = 2-8°C, RT = incubator or air-conditioned room temperature (22-27°C), Incubator = 35±2°C. *SC4 swab = Amies transport swab inoculated at SC1.

**Fig. 2:**
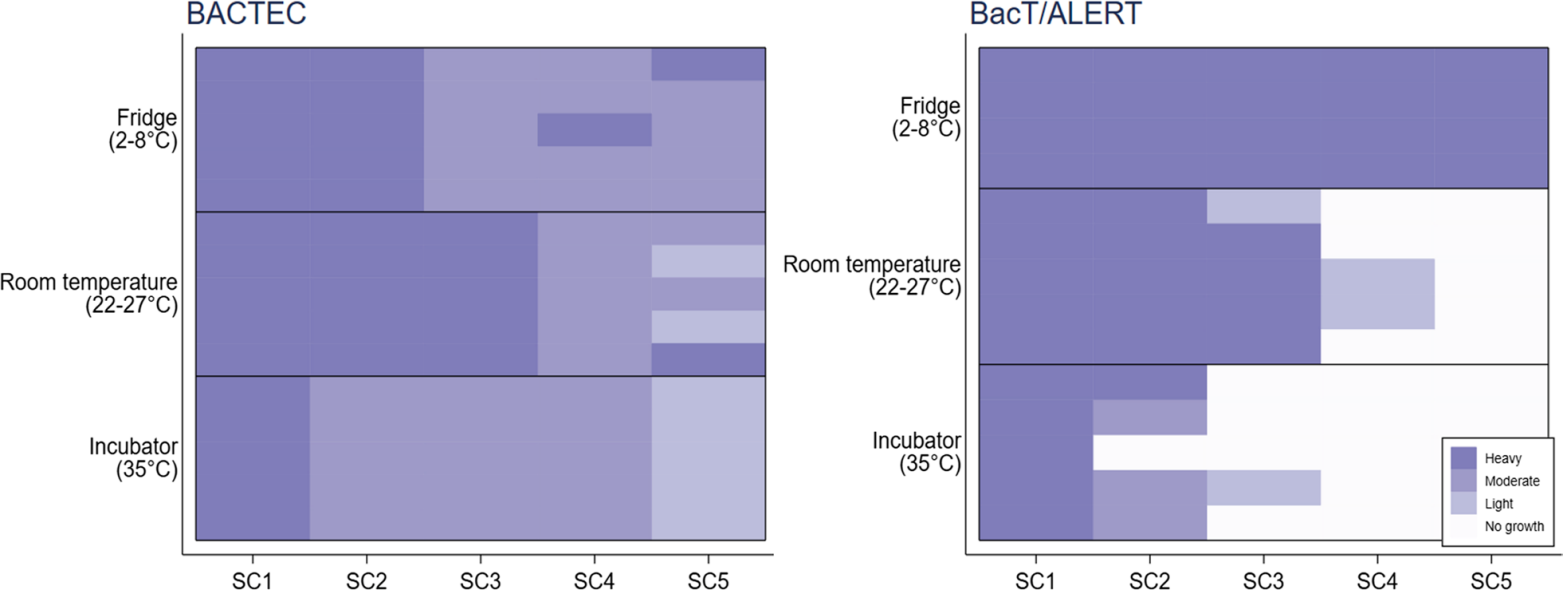
Growth characteristics of *S. pneumoniae* isolates at three different temperature conditions (five isolates per temperature condition, except for Fridge for the BacT/ALERT bottles for which there were four isolates) and sub-culture (SC) time points for the automated blood culture systems BACTEC and BacT/ALERT. SC1 = 0–6 h; SC2 = 22–26 h; SC3 = 30–34 h; SC4 = 46–50 h; SC5 = 7 days

When semi-quantitative results were considered, there was heavy growth (3 + or 4+) of *S. agalactiae*, *S. aureus*, *H. influenzae* and *E. coli* at every time point and for all temperature conditions. For *S. pneumoniae* there was a trend of reduced growth at higher storage temperatures and longer delays to sub-culture for the bottles from both BACTEC and BacT/ALERT systems (Fig. 2).

##### Sub-culture results: amies transport swabs

All organisms grew from the Amies transport swabs stored at every temperature condition with the exception of *H. influenzae*, for which 10/10 swabs stored in the fridge and at RT grew on sub-culture, but only 2/5 swabs stored at 35 ± 2 °C grew when sub-cultured (Table 1). Notably, *S. pneumoniae*, which only grew from 6/14 bottles from the BacT/ALERT system on SC4, was recovered from all (14/14) of the paired Amies transport swabs (SC4 swab).

### Phase two

In total, 125 consecutive positive clinical BC bottle samples were included from the four sites from adults and children between 26 May and 30 July 2021: 50 at LOMWRU, 40 at CPH, 27 at COMRU, and eight at SMRU. The median time to sub-culture was 3 h 30 min (IQR 40 min to 8 h 22 min). There were 140 organisms isolated (51 different species); 13 bottles had mixed growth (Additional file 1: Table S3). All 134 organisms that grew on SC1 grew for all successive sub-cultures, including from the Amies transport swabs (SC4 swab), apart from one *Streptococcus suis* which failed to grow on SC5. The median ambient room temperature for all four sites was 24.4 °C (IQR 23.1–25.6) (Additional file 1: Fig. S1).

When semi-quantitative results were considered, amongst the 134 organisms that grew on SC1, *Streptococcus* spp. resulted in lower quantifications compared with all other organisms combined at SC5 (p = 0.013; Additional file 1: Table S4).

Seven of the 13 bottles with mixed growth grew the same two organisms for all sub-cultures. A third organism was recovered on SC5 for two bottles (*Staphylococcus epidermidis* from a bottle with *Staphylococcus hominis* and *Staphylococcus haemolyticus* on SC1-SC4, and *Brevundimonas vesicularis* from a bottle with *Sphingomonas paucimobilis* and *Micrococcus luteus* on SC1-SC4). For the other four bottles with mixed growth, the second organism did not grow on SC1 but grew on later sub-cultures (one coagulase negative *Staphylococcus* sp. with *Burkholderia* sp. on SC2-SC5, one yeast with *E. coli* on SC2-SC4 and SC4 swab, one *Stenotrophomonas maltophilia* with yeast at SC4, SC4 swab and SC5, and one *E. coli* with *M. luteus* on SC2 only).

Two patients contributed two blood cultures each; the first grew *S. hominis* and *Streptococcus mitis* from the initial BC and *Enterococcus gallinarum* and a coagulase negative *Staphylococcus* sp. from a second BC a week later; the second had two blood cultures collected simultaneously, both of which grew *Candida tropicalis.*

## Discussion

This study investigated the effects of delay to sub-culture at different temperature conditions on the yield of growth for BC bottles that have flagged positive in an automated detection system, in order to simulate the impact on yield of sending positive blood cultures to a central laboratory for further processing. The results show that the majority of blood culture isolates remain viable after seven days of storage at RT (22–27 °C). While previous studies have looked at the effect of time and temperature conditions on organism viability before BC bottles are incubated [2, 3], this study focuses on the effect that time delays and different temperature conditions have once BC bottles have flagged positive. This is important for low-resource and decentralised settings where capacity for on-site organism identification and antimicrobial susceptibility testing may be limited and logistical constraints may prevent immediate transport of bottles that have flagged positive. This study also demonstrates the utility of Amies transport swabs for storing BC broth for easy and safe transport to central laboratories.

There were discrepant results for *S. pneumoniae* between the BACTEC and BacT/ALERT systems with all sub-cultures growing for the bottles from the BACTEC system but only 2/5 bottles with growth at the 48 h sub-culture from the bottles from the BacT/ALERT system stored at RT and no growth after 48 h for the bottles stored at 35 °C. Autolysis of *S. pneumoniae* has previously been described from the BacT/ALERT system [4, 9]. As stored clinical isolates and different PRBCs were used at each site it is also possible that the observed differences could relate to the different strains and blood products, rather than the automated detection systems.

If the facilities exist, performing a Gram stain on positive BCs at the site immediately after bottles flag positive could aid timely clinical treatment and also identify presumptive *S. pneumoniae* so they can be stored in as cool a temperature as practical, prioritised for transport to the reference laboratory, and not assumed to be false-positives if they subsequently fail to grow on sub-culture. The comparison between semi-quantitative growth at SC5 for *Streptococcus* spp. and other organisms suggests that this approach may have benefits for other Streptococci too. Performing a Gram stain, reliable cold storage and/or expediting transport may not be feasible in some settings and it is notable that all *S. pneumoniae* grew from the Amies transport swabs, illustrating their usefulness for preserving viability of *S. pneumoniae* even after 48 h of storage in ambient tropical temperatures. However, it is important to note that this effect may be organism-specific, as *H. influenzae* was not recovered from all Amies transport swabs.

There were six BCs with mixed growth where additional organisms did not grow on SC1 but grew from subsequent sub-cultures. For three of the mixed BCs, one BC had a *Burkholderia* sp. grow from SC2 onwards mixed with a coagulase negative *Staphylococcus*, one had a yeast grow on SC4 onwards in a bottle mixed with *S. maltophilia*, and one had an *E. coli* grow from SC2 onwards that was mixed with a yeast. As all three of these organisms grew from the Amies swabs, it suggests that these organisms were in the original BC broth but either failed to grow from SC1 or were too few to be seen on the agar plate. It is possible that the delayed sub-culture facilitated the detection of these organisms, though we do not suggest routinely delaying or repeating sub-cultures. Routinely delaying sub-cultures would increase time-to-result, whilst repeating sub-cultures may result in a greater number of mixed or contaminated cultures, which may cause difficulties for clinical interpretation and patient management.

There were several limitations to this study. In the real-world BC bottles would be agitated during transit and this was not simulated in this study. This may influence the outcome of the culture results, but to what effect is unclear; agitation may oxygenate the bottle and preserve organism viability; equally, promoting bacterial replication may result in exhaustion of the nutrients available in the culture media leading to premature death. Also, in the real-world ambient temperature would fluctuate more so than in our simulated study. Whilst we stored positive bottles on the laboratory bench during Phase Two, this occurred in air-conditioned laboratories; we felt it was a reasonable assumption that most laboratories using an automated detection system would be temperature-controlled, although air-conditioning units may be turned off at night and temperatures during bottle transport would vary. Unfortunately, it was not possible to cover all possible temperature conditions in this study. For Phase One, individual organisms were only tested at one site and limited to one strain (except for *S. pneumoniae*) and therefore were not inoculated into the same BC bottles. Different manufacturers' bottles and bacterial strains may have an effect on the viability of organisms. For Phase One, PRBCs were used instead of whole blood as this was more accessible to all the laboratories. Pilot experiments from one of the sites showed no difference between organism growth and bottle positivity using PRBCs compared to whole blood (unpublished data). Finally, there were no clinical BC bottles that grew *S. pneumoniae* or *H. influenzae* in Phase Two. As these organisms had varying growth results in Phase One, it would have been interesting to see how these organisms grew in Phase Two from clinical samples. Although we focused on five organisms for Phase One, the inclusion of consecutive clinical samples from four different sites for Phase Two meant a larger variety of organisms were included and all grew on all sub-cultures except one *S. suis*.

Despite these limitations, this study illustrates how temperature can effect organism viability and demonstrates the utility of Amies transport swabs for storing BC broth for easy transportation. The strength of this study is that it was replicated in four different laboratory locations and included a diverse number of organisms. The testing of three different temperature conditions at four different time points allowed us to show the longitudinal impact of the different temperatures and time delays.

## Conclusions

This study shows that the majority of organisms from BC bottles that have flagged positive in an automated detection system can be stored and transported at room temperature (22–27 °C) without negatively influencing organism viability. Delays to sub-culture should always be minimised but, when unavoidable, storing positive BC bottles in as cool a temperature as feasible, the use of Amies transport swabs and performing a Gram stain prior to transport may improve results, particularly for organisms susceptible to autolysis, such as *S. pneumoniae*. However, the use of transport swabs might not be a viable option for *H. influenzae*. These findings are relevant for low-resource and decentralised settings where positive BC bottles may not be processed immediately and delays to sub-culture are unavoidable. Further research is needed to investigate the impact of temperature conditions and time delays for other blood culture systems (e.g. manual) and organisms not included in this study.

## Supplementary Information

**Additional  file 1: Table S1. **Median inoculum, time to positivity, time to removal from the automated detection system, and time to SC1, for each of the target organisms. IQR = inter-quartile range; SC1 = sub-culture 1. *after removal from the machine, **after flagging positive. **Table S2**. Wilcoxon matched-pairs signed-rank test p-values for the comparison of growth of *Streptococcus pneumoniae* from Phase One for all bottles (i.e., BACTEC and BacT/ALERT combined). Comparisons are between SC1 versus all other SCs, and SC4 versus SC4 swab, within each temperature condition. *All bottles resulted in growth. SC1 = 0–6 h; SC2 = 22–26 h; SC3 = 30–34 h; SC4 = 46–50 h; SC4 swab = Amies transport swab stored for 46–50 h; SC5 = 7 days. Values in bold indicate p < 0.05. **Table S3**. Organisms isolated from positive clinical blood culture bottles from the four clinical sites and between May and July 2021. Twenty-eight organisms were isolated from 13 mixed blood cultures. **Table S4**. Comparison of semi-quantitative growth of 11 isolates of *Streptococcus* spp. compared to 123 other organisms at each sub-culture time point for Phase Two, using Fisher’s exact test. *3 degrees of freedom. SC1 = 0–6 h; SC2 = 22–26 h; SC3 = 30–34 h; SC4 = 46–50 h; SC4 swab = Amies transport swab stored for 46–50 h; SC5 = 7 days. Values in bold indicate p < 0.05. **Fig. S1.** Ambient temperature at the four microbiology laboratories during Phase 2. Median temperature across all four sites was 24.4 °C (IQR 23.1–25.6), at CPH 23.5 °C (23.1–23.8), COMRU 24.7 °C (24.0-25.6), LOMWRU 25.7 °C (25.3–25.9) and SMRU 22.1 °C (21.7–22.5). CPH recorded temperatures manually three times per day for the first two weeks (6 July to 19 July 2021, data not shown); median temperature was 24.7 °C (IQR 24.2–25.2).

## Data Availability

The dataset supporting the conclusions of this article and the additional files are available in the Figshare repository, DOI: 10.6084/m9.figshare.17089514.v3.

## Abbreviations

BC: Blood culture
BSI: Blood stream infection
COMRU: Cambodia Oxford Medical Research Unit
SMRU: Shoklo Malaria Research Unit
CPH: Chiangrai Prachanukroh Hospital
LMIC: Low- and middle- income country
LOMWRU: Lao-Oxford-Mahosot Hospital-Wellcome Trust Research Unit
PRBC: Packed red blood cells
RT: Room temperature
