# Supplementary material for: Impact of delayed processing of positive blood cultures on organism detection: a prospective multi-centre study
Source: BMC Infect Dis. 2022 Jun 4;22:517. doi: 10.1186/s12879-022-07504-1 (PMC9167519; doi:10.1186/s12879-022-07504-1)
Supplement: Supplementary file 1 — Additional file 1: Table S1. Median inoculum, time to positivity, time to removal from the automated detection system, and time to SC1, for each of the target organisms. IQR = inter-quartile range; SC1 = sub-culture 1. *after removal from the machine, **after flagging positive. Table S2. Wilcoxon matched-pairs signed-rank test p-values for the comparison of growth of Streptococcus pneumoniae from Phase One for all bottles (i.e., BACTEC and BacT/ALERT combined). Comparisons are between SC1 versus all other SCs, and SC4 versus SC4 swab, within each temperature condition. *All bottles resulted in growth. SC1 = 0–6 h; SC2 = 22–26 h; SC3 = 30–34 h; SC4 = 46–50 h; SC4 swab = Amies transport swab stored for 46–50 h; SC5 = 7 days. Values in bold indicate p < 0.05. Table S3. Organisms isolated from positive clinical blood culture bottles from the four clinical sites and between May and July 2021. Twenty-eight organisms were isolated from 13 mixed blood cultures. Table S4. Comparison of semi-quantitative growth of 11 isolates of Streptococcus spp. compared to 123 other organisms at each sub-culture time point for Phase Two, using Fisher’s exact test. *3 degrees of freedom. SC1 = 0–6 h; SC2 = 22–26 h; SC3 = 30–34 h; SC4 = 46–50 h; SC4 swab = Amies transport swab stored for 46–50 h; SC5 = 7 days. Values in bold indicate p < 0.05. Fig. S1. Ambient temperature at the four microbiology laboratories during Phase 2. Median temperature across all four sites was 24.4 °C (IQR 23.1–25.6), at CPH 23.5 °C (23.1–23.8), COMRU 24.7 °C (24.0-25.6), LOMWRU 25.7 °C (25.3–25.9) and SMRU 22.1 °C (21.7–22.5). CPH recorded temperatures manually three times per day for the first two weeks (6 July to 19 July 2021, data not shown); median temperature was 24.7 °C (IQR 24.2–25.2). [file 12879_2022_7504_MOESM1_ESM.docx]

Impact of delayed processing of positive blood cultures on organism detection: a prospective multi-centre study

**Additional files**

**Additional Table 1**. Median inoculum, time to positivity, time to removal from the automated detection system, and time to SC1, for each of the target organisms. IQR = inter-quartile range; SC1 = sub-culture 1. * after removal from the machine, **after flagging positive

| **Organism**  **(automated detection system, laboratory site)** | **Median CFU/ml** | **Median time to positivity (IQR)** | **Median time to removal from machine**  **(IQR)** | **Median time to SC1 (IQR)*** | **Median total time to SC1****  **(IQR)** |
| --- | --- | --- | --- | --- | --- |
| ***S. agalactiae***  (BACTEC, LOMWRU) | 7.0 | 9 h 18 min  (9 h 18 min – 9 h 28 min) | 8 h 29 min  (8 h 19 min – 8 h 29 min) | 1 h 00 min  (1 h 00 min – 1 h 00 min) | 9 h 29 min  (9 h 19 min – 9 h 29 min) |
| ***S. pneumoniae***  (BACTEC, LOMWRU) | 7.4 | 12 h 30 min  (12 h 15 min – 12 h 50 min) | 5 h 17 min  (4 h 57 min – 5 h 31 min) | 0 h 30 min  (0 h 30 min – 0 h 30 min) | 5 h 47 min  (5 h 27 min – 6 h 2 min) |
| ***S. pneumoniae***  (BacT/ALERT, COMRU) | 4.5 | 17 h 28 min  (17 h 15 min – 17 h 40 min) | 0 h 0 min  (0 h 0 min – 0 h 2 min) | 0 h 5 min  (0 h 5 min – 0 h 5 min) | 0 h 5 min  (0 h 5 min – 0 h 7 min) |
| ***S. aureus***  (BacT/ALERT, SMRU) | 10.6 | 13 h 3 min  (12 h 50 min – 13 h 11 min) | 4 h 40 min  (4 h 30 min – 4 h 55 min) | 0 h 30 min  (0 h 26 min – 0 h 40 min) | 5 h 11 min  (5 h 3 min – 5 h 25 min) |
| ***H. influenzae***  (BacT/ALERT, COMRU) | 1.2 | 19 h 48 min  (19 h 32 min – 20 h 14 min) | 0 h 0 min  (0 h 0 min – 0 h 0 min) | 0 h 28 min  (0 h 19 min – 0 h 34 min) | 0 h 28 min  (0 h 19 min – 0 h 34 min) |
| ***E. coli***  (BacT/ALERT, COMRU) | 4.0 | 12 h 49 min  (12 h 37 min – 12 h 52 min) | 2 h 30 min  (2 h 29 min – 2 h 40 min) | 2 h 20 min  (2 h 20 min – 2 h 20 min) | 4 h 50 min  (4 h 49 min – 5 h 0 min) |

**Additional Table 2**. Wilcoxon matched-pairs signed-rank test p-values for the comparison of growth of *Streptococcus pneumoniae* from Phase One for all bottles (i.e., BACTEC and BacT/ALERT combined). Comparisons are between SC1 versus all other SCs, and SC4 versus SC4 swab, within each temperature condition. *All bottles resulted in growth. SC1 = 0-6 h; SC2 = 22-26 h; SC3 = 30-34 h; SC4 = 46-50 h; SC4 swab = Amies transport swab stored for 46-50 h; SC5 = 7 days. Values in **bold** indicate p <0.05.

| **Sub-culture** | **Wilcoxon matched-pairs signed-rank exact p-value** | | |
| --- | --- | --- | --- |
|  | **Fridge** | **Room temperature** | **Incubator** |
| **SC1 compared to:** | | | |
| SC2 | 1.000* | 1.000* | **0.004** |
| SC3 | 0.063 | 1.000 | **0.002** |
| SC4 | 0.125 | **0.002** | **0.002** |
| SC4 swab | 0.063 | 0.063 | **0.016** |
| SC5 | 0.125 | **0.004** | **0.002** |
| **SC4 compared to SC4 swab** | 1.000* | 0.063 | 0.221 |

**Additional Table 3**. Organisms isolated from positive clinical blood culture bottles from the four clinical sites and between May and July 2021. Twenty-eight organisms were isolated from 13 mixed blood cultures.

| **Organism type** | **Organism** | **Number of isolates per clinical site (automated detection system)** | | | | |
| --- | --- | --- | --- | --- | --- | --- |
|  |  | **LOMWRU**  **(BACTEC)** | **COMRU**  **(BacT/ALERT)** | **SMRU**  **(BacT/ALERT)** | **CPH**  **(BACTEC)** | **Total** |
| **Gram-negative** | *Acinetobacter baumannii* | 1 | 2 | 0 | 1 | **4** |
|  | *Acinetobacter lwoffi* | 2 | 0 | 0 | 0 | **2** |
|  | *Brevundimonas vesicularis* | 0 | 1 | 0 | 0 | **1** |
|  | *Burkholderia cepacia* | 1 | 0 | 0 | 1 | **2** |
|  | *Burkholderia pseudomallei* | 11 | 0 | 0 | 0 | **11** |
|  | *Burkholderia* spp. | 1 | 0 | 0 | 0 | **1** |
|  | *Edwardsiella tarda* | 0 | 1 | 0 | 0 | **1** |
|  | *Enterobacter cloacae* | 0 | 0 | 0 | 2 | **2** |
|  | *Enterobacter* spp. | 2 | 0 | 0 | 0 | **2** |
|  | *Escherichia coli* | 6 | 4 | 0 | 12 | **22** |
|  | GNR no ID | 1 | 0 | 0 | 0 | **1** |
|  | *Klebsiella pneumoniae* | 6 | 2 | 0 | 3 | **11** |
|  | *Proteus mirabilis* | 0 | 0 | 1 | 1 | **2** |
|  | *Pseudomonas aeruginosa* | 3 | 0 | 0 | 4 | **7** |
|  | *Pseudomonas* sp. | 0 | 0 | 0 | 2 | **2** |
|  | *Rothia muciaginosa* | 0 | 1 | 0 | 0 | **1** |
|  | *Salmonella* Group B | 1 | 0 | 0 | 1 | **2** |
|  | *Salmonella enterica* ssp.*enterica* | 0 | 1 | 1 | 0 | **2** |
|  | *Salmonella* spp. | 0 | 1 | 0 | 0 | **1** |
|  | *Sphingomonas paucimobilis* | 0 | 1 | 0 | 0 | **1** |
|  | *Stenotrophomonas maltophilia* | 1 | 0 | 0 | 1 | **2** |
| **Gram-positive** | *Bacillus firmus* | 0 | 0 | 1 | 0 | **1** |
|  | *Bacillus* spp. | 0 | 0 | 0 | 1 | **1** |
|  | *Corynebacterium amycolatum* | 0 | 0 | 1 | 0 | **1** |
|  | *Corynebacterium aurimucosum* | 0 | 1 | 0 | 0 | **1** |
|  | *Corynebacterium* spp. | 0 | 0 | 0 | 2 | **2** |
|  | *Diptheroids* | 1 | 0 | 0 | 0 | **1** |
|  | *Enterococcus faecalis* | 1 | 0 | 0 | 1 | **2** |
|  | *Enterococcus faecium* | 0 | 0 | 0 | 2 | **2** |
|  | *Enterococcus gallinarium* | 2 | 0 | 0 | 0 | **2** |
|  | *Microbacterium trichothecenolyticum* | 0 | 2 | 0 | 0 | **2** |
|  | *Micrococcus luteus* | 0 | 2 | 0 | 1 | **3** |
|  | *Micrococcus* spp. | 2 | 0 | 0 | 0 | **2** |
|  | *Staphylococcus arlettae* | 0 | 1 | 0 | 0 | **1** |
|  | *Staphylococcus aureus* | 2 | 0 | 1 | 1 | **4** |
|  | *Staphylococcus capitis* | 0 | 0 | 1 | 0 | **1** |
|  | *Staphylococcus epidermidis* | 1 | 2 | 0 | 0 | **3** |
|  | *Staphylococcus haemolyticus* | 0 | 2 | 0 | 0 | **2** |
|  | *Staphylococcus hominis* | 1 | 2 | 1 | 0 | **4** |
|  | *Staphylococcus sciuri* | 0 | 0 | 0 | 1 | **1** |
|  | *Staphylococcus warneri* | 0 | 0 | 0 | 1 | **1** |
|  | Coagulase negative *Staphylococcus* spp. | 7 | 0 | 0 | 0 | **7** |
|  | *Streptococcus gallolyticus* | 0 | 0 | 1 | 0 | **1** |
|  | *Streptococcus gordoni* | 0 | 0 | 0 | 1 | **1** |
|  | *Streptococcus* Group A/ *pyogenes* | 0 | 3 | 0 | 1 | **4** |
|  | *Streptococcus intermedius* | 1 | 0 | 0 | 0 | **1** |
|  | *Streptococcus mitis* | 1 | 0 | 0 | 1 | **2** |
|  | *Streptococcus suis* | 1 | 0 | 0 | 1 | **2** |
|  | *Candida tropicalis* | 0 | 2 | 0 | 0 | **2** |
| **Yeast** | Yeast | 0 | 0 | 0 | 2 | **2** |
|  | *Cryptococcus neoformans* | 1 | 0 | 0 | 0 | **1** |
|  | Mixed bottles | 7 | 2 | 0 | 4 | **13** |
|  | **TOTAL ORGANISMS** | **57** | **31** | **8** | **44** | **140** |
|  | **TOTAL BOTTLES** | **50** | **27** | **8** | **40** | **125** |


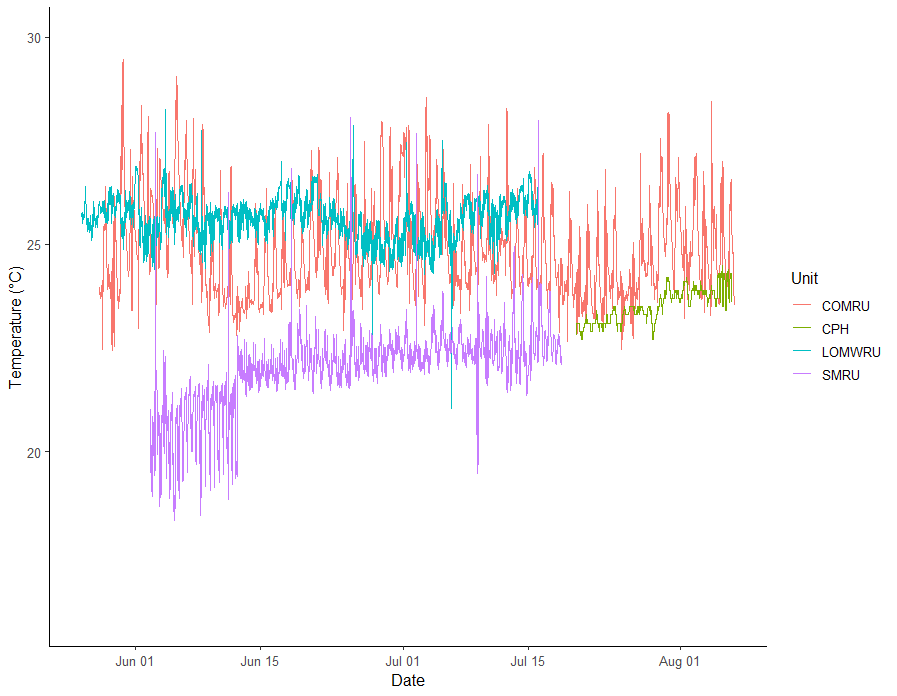


**Additional Figure 1.** Ambient temperature at the four microbiology laboratories during Phase 2. Median temperature across all four sites was 24.4°C (IQR 23.1-25.6), at CPH 23.5°C (23.1-23.8), COMRU 24.7°C (24.0-25.6), LOMWRU 25.7°C (25.3-25.9) and SMRU 22.1°C (21.7-22.5). CPH recorded temperatures manually three times per day for the first two weeks (6 July to 19 July 2021, data not shown); median temperature was 24.7°C (IQR 24.2-25.2).

**Additional Table 4**. Comparison of semi-quantitative growth of 11 isolates of *Streptococcus* spp. compared to 123 other organisms at each sub-culture time point for Phase Two, using Fisher’s exact test. **3 degrees of freedom*. SC1 = 0-6 h; SC2 = 22-26 h; SC3 = 30-34 h; SC4 = 46-50 h; SC4 swab = Amies transport swab stored for 46-50 h; SC5 = 7 days. Values in **bold** indicate p <0.05.

| **Sub-culture** | **Fisher’s exact p-value*** |
| --- | --- |
| **SC1** | 0.120 |
| **SC2** | 0.115 |
| **SC3** | 0.139 |
| **SC4** | 1.000 |
| **SC4 swab** | 0.269 |
| **SC5** | **0.013** |
